# Severe pertussis in a young infant due to household transmission: the needs of pertussis vaccination boosters in Japan

**DOI:** 10.1002/ccr3.1472

**Published:** 2018-03-08

**Authors:** Daiji Takajo, Shigeaki Nonoyama

**Affiliations:** ^1^ Department of Pediatrics National Defense Medical College 3‐2 Namiki Tokorozawa Saitama 359‐8513 Japan

**Keywords:** Booster, infant, LAMP, loop‐mediated isothermal amplification, pertussis, vaccine

## Abstract

Household is responsible for a large percentage of pertussis infection in young infants. Japanese vaccine recommendation committee does not recommend any boosters for teens and pregnant women. Because of its high vaccine effectiveness, introduction of vaccination for pregnant women is high priority to prevent pertussis infection in young infants.

## Introduction

Neonatal and very young infants with pertussis often show severe clinical course with apnea, seizures, and encephalopathy, resulting in the highest mortality among all age group. Avoiding transmission to this age group should be strongly encouraged. Centers for Disease Control and Prevention (CDC) recommends pertussis vaccination for all babies and children, preteens and teens, and pregnant women. However, Japanese immunization program does not recommend additional boosters for adolescents, pregnant women, and adults. Here we report a 35‐day‐old Japanese infant who was infected with severe pertussis via household transmission, her mother, and siblings. The patient had apneic, bradycardic episodes, and required intensive care. Administration of appropriate boosters for adolescents and adults, especially pregnant women, is a public health concern.

## Case Presentation

A 35‐day‐old Japanese infant with no past medical history presented with 2 days of cough, cyanosis, and apnea. Mother noticed loss of energy and posttussive emesis. Mother also described the patient experienced apneic episodes for several seconds but denied any other symptoms. The patient was full term, was born by cesarean delivery, and weighed 2760 g at birth. The pregnancy was uncomplicated. She was on Japanese immunization schedule and had not received any vaccination at the time of admission. The patient had no sick contacts except for her mother, her eight‐year‐old brother, and six‐year‐old sister all of who had cough without any doctor's visits. Her siblings and mother received DPT vaccination three times during infancy, followed by fourth injection at age one. Mother did not receive a pertussis boost during pregnancy. None of them were administered booster vaccine for pertussis.

On physical examination, the patient showed increased work of breathing with decreased energy. She had paroxysms of whooping cough with reddened face. Vital signs were temperature 36.6‐degree, pulse rate 168, respiratory rate 24, and blood pressure 77/46 mmHg. She was saturated 90–95% in room air without apnea, but the number dropped down to 70% with apnea. Otherwise, her physical exams were unremarkable.

Laboratory data shows white blood cell count 14,000/uL with 51.6% lymphocyte (absolute lymphocyte count 7224/uL), negative for C‐reactive protein level, and lactate 12.0 mg/dL. She had severe respiratory acidosis with pH 7.27, pCO2 58.6 mmHg, HCO3‐ 25.8 mmHg in venous blood gas. Serum anti‐pertussis toxin (PT) IgG antibody was negative. Otherwise, blood test was unremarkable.

On admission, she was intubated due to frequent apneic episodes, and intravenous antibiotics were started after intratracheal aspirates was obtained for culture and a LAMP assay for pertussis. Azithromycin 12 mg/kg/day was administered for suspicion of pertussis and cefotaxime 100 mg/kg/day was for suspicion of sepsis. Cardiac echocardiogram revealed no signs of pulmonary hypertension or right heart failure. Over the next 4 days after admission, bradycardic episodes had occurred frequently, and some episodes required breathing assist. The diagnosis of pertussis was made on day three of admission when the result of a pertussis LAMP assay came back positive. Early diagnosis and intensive care were provided, and the patient discharged on day 17 of admission without any neurological complications.

## Discussion

The incidence of pertussis is highest in infants, with mortality rates greatest in infants younger than 3 months 1. Prevention of transmission to infants of this age group is a critical public health issue. However, the efforts to prevent pertussis are not fully taken in Japan yet. Against recommendation by CDC, Japanese vaccination schedule includes only four‐time shots during infancy without any additional boosters. This may contribute to a higher pertussis prevalence among adults, but the exact prevalence is still unknown.

### Pertussis in Japan 2

Clinically diagnosed pertussis cases are reported weekly from approximately 3000 pediatric sentinel sites under the National Epidemiological Surveillance of Infectious Diseases system.

The annual number of reported cases per sentinel site was ranging 0.53–2.24 in the last decade, and 0.95 in 2016 (2989 cases in all sentinel sites). In 2016, 9.5% of the cases were 0–5 months old, 3.5% were 6–11 months. Although reporting comes from pediatric sentinel sites, there are reported patients older than 15 years old; reports among this age group were 0.24 in 2016, occupying 25% of all pertussis patients. Epidemiological studies of adult pertussis have not fully conducted in Japan. Pertussis immunization schedule is completed with four doses of shots; three doses given with intervals at least 20 days apart (the recommended interval is 20–56 days) starting at 3 months of age, followed by one shot given at least 6 months after the third dose. No additional boosters for teens and adults are recommended. Seroprevalence survey was conducted in 2013 and revealed anti‐PT IgG antibodies were positive in 90% of infants aged 6–11 months, 30% of children aged 5–6 years. Positive rate increases thereafter, indicated natural infections.

### Pertussis in other countries

Boosters for teens, preteens, and pregnant women are recommended by CDC. In the United States, five doses of DTaP are recommended between 6 weeks and 7 years, followed by a Tdap booster dose for 11–12 years 3. One more additional booster is recommended for adults 3. Similar vaccination schedule is recommended in Canada, Germany, and other many European countries 4, 5. A systematic literature view revealed the average duration of vaccine protection from DTaP is estimated to be ~3 years and concluded very few children over age 10 would be protected against pertussis 6. Multiple epidemiological studies also demonstrated that pertussis vaccination during each pregnancy can reduce pertussis infection in infants and medical cost 7, 8, 9. In a study in England, maternal vaccination at least 7 days before birth shows 91% of vaccine effectiveness regarding pertussis infection in infants under the age of 3 months 7. Moreover, vaccine effectiveness drops to 38% if maternal vaccine is provided 0–6 days before or 1–13 days after birth 7. A prospective international multicenter study showed household members were responsible for 76–83% of transmission of *B. pertussis* 10. Thus, pertussis boosters for adolescents and adults, especially for pregnant women, are important to protect severe pertussis among infants.

### Case review

In our case, a young infant without the first dose of pertussis vaccination was infected from household. Her siblings and mother completed four doses of pertussis vaccination, and no additional booster vaccines for pertussis were administered. Her siblings (eight‐year‐old brother and six‐year‐old sister) were in the age group, which seemed to be in the lowest anti‐PT IgG antibody positive rate 2. The patient had a severe clinical course with apnea and bradycardia, requiring 10 days of PICU stay. Fortunately, she recovered completely without any neurological complications. It is reported that admission to intensive care unit, premature birth, and vaccination status are related to higher cost for medical care 11. Thus, prevention and early diagnosis/interventions are essential. Early diagnosis was made in this case with a commercial loop‐mediated isothermal amplification (LAMP) assay, which was developed in 2015 in Japan 12. Although polymerase chain reaction (PCR) is commonly used as laboratory confirmation worldwide, a LAMP assay is more useful because isothermal amplification is carried out at a constant temperature and does not require a thermal cycler 13. This commercial diagnosis kit may contribute to early diagnosis of pertussis and further investigation of epidemiology.

## Conclusion

Preventing household transmission of pertussis is important to prevent infants from severe pertussis infection. Because of its high vaccine effectiveness, pertussis vaccination boosters for pregnant women should be introduced in Japan with the highest priority.

## Conflict of Interest

The authors declare no conflict of interests.

## Authorship

DT: drafted the manuscript. SN: revised and approved the manuscript.
